# Scientific and Regulatory Standards for Assessing Product Performance Using the Similarity Factor, *f2*

**DOI:** 10.1208/s12248-015-9723-y

**Published:** 2015-02-12

**Authors:** Ruth E. Stevens, Vivian Gray, Angelica Dorantes, Lynn Gold, Loan Pham

**Affiliations:** 1 Camargo Pharmaceutical Services, 9825 Kenwood Road, Cincinnati, OH 45242 USA; 2 V.A. Gray Consulting, Hockessin, DE 19707 USA; 3 Food and Drug Administration, White Oak Campus, Silver Spring, MD 20993 USA

**Keywords:** *f*2, similarity factor, 505(b)(2), dissolution, bootstrap

## Abstract

The similarity factor, *f*2, measures the sameness of dissolution profiles. The following commentary is an overview of discussions and presentations from a group of industry and US regulatory experts that have integrated the science and regulatory research and practice for assessing product performance, particularly for modified-release (MR) dosage forms, using *f*2. For a drug development sponsor or applicant with an orally complex dosage formulation, it is critical to understand dissolution methods and the similarity factor and how and/or when to apply it in their NDA, ANDA, or PMA submission. As part of any regulatory submission, it is critical to justify that the product performance has not been impacted by any change in the manufacturing process and/or the delayed and/or prolonged drug release characteristics compared to a similar conventional or another orally complex dosage form. The purposes of this document are (1) to provide a description of appropriate dissolution methods, how is the *f*2 calculated and how it can be used to justify product performance similarity, or not; (2) to provide an overview of alternative methods available for dissolution profile comparisons, and (3) to illustrate how applying these concepts in a focused way supports approval of submissions and regulatory dossiers and aligns them with on-going science and regulatory initiatives. A case study will be used as an example to demonstrate how dissolution testing and the *f*2 calculation results can impact regulatory outcomes from an NDA (505(b)(1)), NDA (505(b)(2)), ANDA (505(j)), supplemental NDAs/ANDAs, or PMA perspective.

## BACKGROUND INFORMATION

The authors of this paper were selected to present a roundtable presentation entitled, “Scientific and Regulatory Standards for Assessing Product Performance Using the Similarity Factor, *f2*” at 2013 American Association of Pharmaceutical Scientists (AAPS) Annual Meeting and Exposition which took place November 10–14, 2013 in San Antonio, Texas. The moderators of the roundtable were Lynn Gold, PhD and Loan Pham, PhD. Vivian Gray presented the topic, “Scientific Considerations Using the Similarity Factor, *f2*”. Ruth Stevens, PhD, MBA presented the topic, “Practical Applications of the Similarity Factor, *f2*”, followed by Angelica Dorantes, PhD, who presented “Regulatory Applications of *f2*”. The authors were invited by the Scientific Journal of the AAPS to submit their combined presentations as a commentary to this journal for wider distribution among its community.

There is a growing recognition of the importance of submitting a quality regulatory submission, especially when FDA refuses to file an application under 21 CFR314.101(d)(3) because the application or abbreviated application is incomplete (does not on its face contain information required under section 505(b) and 21 CFR 314.50). This is what happened to Merck & Co when they first submitted their NDA, ezetimibe/atorvastatin tablets. Pertinent to this commentary, two out of the four reasons quoted for the filing deficiency were as follows: (1) “The primary stability batches were manufactured at a Research and Development (R&D) facility. Provide stability data to bridge the R&D manufacturing to the commercial manufacturing (i.e., data for three commercial batches with at least three months of long term and accelerated data) as well as multipoint dissolution profiles.” and (2) “The application did not include any information to bridge the performance of the clinically tested batches to the commercial products (e.g., multipoint *in vitro* dissolution profiles)” (1).

This paper will discuss further how the similarity *f*2 statistical comparison was used for the reformulated Asacol® capsule product, not as a point estimator but in combination with a statistical model independent multivariate confidence region procedure (Bootstrap approach), where wide variability was observed in the dissolution profiles. It will also briefly discuss alternative statistical approaches that may be considered when application of the *f* 2 metric is not appropriate.

## SCIENTIFIC CONSIDERATIONS USING THE SIMILARITY FACTOR, *F*2

Information on *f*2 can be found in at least three guidances: Guidance for Industry, Dissolution Testing of Immediate Release Solid Oral Dosage Forms (2), Guidance for Industry, Waiver of *In Vivo* Bioavailability and Bioequivalence Studies for Immediate Release Solid Oral Dosage Forms Based on the a Biopharmaceutics Classification System (3), and Guidance for Industry Immediate Release Solid Oral Dosage Forms, Scale-Up and Post-Approval Changes: Chemistry, Manufacturing Controls, *In Vitro* Dissolution Testing and *In Vivo* Bioequivalence Documentation (4). These three guidances have essentially the same information but the most extensive treatment of the subject is from the Guidance for Industry, Dissolution Testing of Immediate Release Solid Oral Dosage Forms (Table I).

**Table I:** FDA Guidances for *f*2

Time points	Variability	Sample units	Measurements
Three to four or more	RSD NMT 20% for earlier time point (e. g. 15 min)^a^	Same dissolution conditions for both reference and test product	Use mean value of 12 units for each reference and test product
Same for both reference (prechange) and test product	RSD NMT 10% for the other time points	Reference batch should be most recently manufactured	Only one measurement after 85% of both products

^a^The example is 10 min in the Guidance for Industry, Waiver of *In Vivo* Bioavailability and Bioequivalence Studies for Immediate Release Solid Oral Dosage Forms Based on the a Biopharmaceutics Classification System

Dissolution profile comparisons, in general, refer to the comparison of two dissolution profiles such as that of reference and test batches, pre-change batch and post-change batch, and strengths of product to support a biowaiver for strengths not used in an *in vivo* bioequivalence trial. During method development, there are basic attributes that the method should have to yield appropriate data for *f*2 comparisons. These attributes include low variability, an identifiable slope in the percent drug dissolved over time, minimized artifacts that can bias the shape of these slopes, and an ability to detect changes in product critical quality attributes (CQAs). Furthermore, an understanding of the release mechanism(s) and linkage to clinical outcome are imperative for obtaining a meaningful and “clinically relevant” dissolution method.

When developing a dissolution method, the variability of the data should be monitored and minimized as much as possible. Within any given formulation, high between-unit variability will negatively impact the discriminatory power of this test. Variability may be inherent to the dissolution behavior of the formulations or it may be an artifact of the dissolution method, e.g., coning, tablet landing and sticking to an off-center position under the paddle, capsule pellicle formation, spinning of the dosage unit, and a variety of other clearly noticeable dissolution anomalies (5). All six dissolution vessels should be observed to determine if the anomalies are consistent in all six vessels or only observed in 1 or 2 of the vessels.

A practical consideration has been the implementation of variability constrains when conducting a profile comparison using the *f*2 metric. The relative standard deviation (RSD) should be not more than (NMT) 20% at time points less than or equal to 15 min and the RSD should be NMT 10% for all other points. One could argue that these rules on variability are really quite generous. However, when earlier time points are needed for the *f*2 calculations, this can be a problem. Five-minute time points are needed when rapid disintegration is taking place, therefore variability is expected.

The *f*2 rules dictate that the number of dosage units should be 12 of both of the reference and test or post-change drug products. At least three time points are required, and only one dissolution value above 85% can be used in the calculation (2,6–8). The similarity factor, *f2* is not needed when greater than 85% of the labeled amount of drug in the drug products has been dissolved within 15 min when tested in 0.1 N HCl, pH 4.5 acetate buffer, and pH 6.8 phosphate buffer.

One challenge with existing criteria is that the *in vivo* release may be multiphasic, thereby limiting the ability of the *f*2 metric to predict *in vivo* product. For example, if an oral product was developed to be locally acting and is designed to be intact until at least pH 6.8 or greater (reaches the distal GI tract) and then a burst of drug (greater than 80%) is released in 15–30 min after reaching the site, one could argue that this profile would be similar to that of an immediate-release product dissolution and that the *f*2 rules would be applicable. It might make sense to set the criteria as >85% dissolved in 60 or 90 min at pH 6.8 phosphate buffer or higher and zero dissolution when tested in 0.1 N HCl and pH 4.5 acetate buffer rather than calculate an *f*2 value. With this type of release mechanism, it may be best to employ a risk-based approach when setting the *in vitro* release criteria. However, *in vitro* release tests tend to be completed within 60–90 min. This raises the question of whether or not the existing rules for *f*2 remain applicable. Given the significant advances in drug delivery technology and delivery, further discussion is needed around this point.

## STATISTICAL APPROACHES WHEN *F*2 CANNOT BE APPLIED

When variability exceeds the *f*2 rules, other statistical models should be considered. The following is an overview of two alternative approaches that may be considered, model independent or model dependent methods (2).

### Model Independent Approaches

One such model is the independent multivariate confidence region procedure (Bootstrap approach) method. Bootstrap allows for the use of *f*2, not as a point estimator but as a confidence interval, thus overcoming concerns encountered when *f*2 is used solely as a point estimate. Computation of the bias corrected and accelerated (BCA) confidence intervals is suggested. The bootstrap method simulates the distribution of *f*2 values to determine whether or not two profiles exhibit comparable *in vitro* behavior. (6,9) This can include the following steps:

1. Generate N bootstrap samples (e.g., *N* = 1000) by resampling independently with replacement from dissolution data for the test and reference groups.
2. Calculate *f*2 values from N bootstrap samples.
3. Calculate the bias correction statistic to correct for the potential skewed distribution of f2 derived from the bootstrap samples. This can be achieved by calculating the acceleration statistic (i.e., the rate of change of the standard error of the estimate of the sample statistic) by obtaining n jackknife samples (e.g., *n* = 24) derived from original dissolution data.
4. Calculate lower and upper bounds of the confidence interval using type 1 error, *f*2 values of N bootstrap samples, bias correction, and acceleration statistics.

**Fig. 1:**
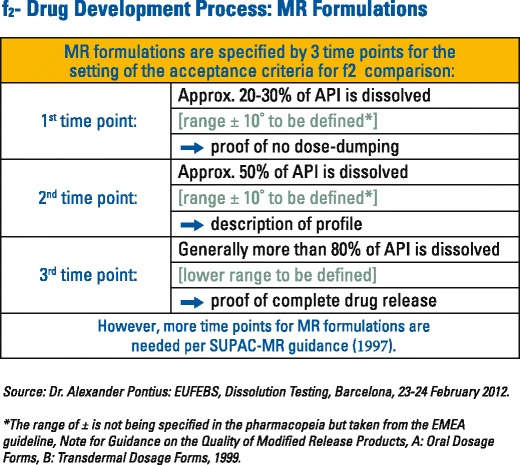
Modified-release dissolution time points

To declare similarity between test and reference dissolution profiles, the lower bound of the confidence interval must equal 50 or more. To illustrate, the bootstrap method was applied by the FDA when they evaluated the reformulated 400 mg mesalamine delayed-release (MDR) (Delzicol^TM^) against the original 400 mg mesalamine modified-release tablets (10). The newly marketed MDR capsule and discontinued tablet (Asacol®) formulations have a pH-dependent release mechanism designed to delay release until the formulation reaches the distal gastrointestinal (GI) tract (pH~6.5), thus, increasing the level of exposure to the colon in patients with ulcerative colitis. The dissolution characteristics and pharmacokinetics of these MDR capsules and tablets are highly variable. As part of the reformulation program to market the capsule formulation, the similarity of dissolution profiles to the original MDR tablet formulation were needed across various pH values to provide the necessary assurance of comparable product performance (10). For modified-release oral dosage forms such as Delzicol^TM^ that are developed for targeted local drug delivery within the GI tract, the dissolution profiles must contain at least three timepoints (11). As stated in the SUPAC MR 1997 guidance, “Adequate sampling should be performed, for example, at 1, 2, and 4 h and every two hours thereafter until either 80% of the drug from the drug product is released or an asymptote is reached.” (12) (Fig. 1).

**Fig. 2:**
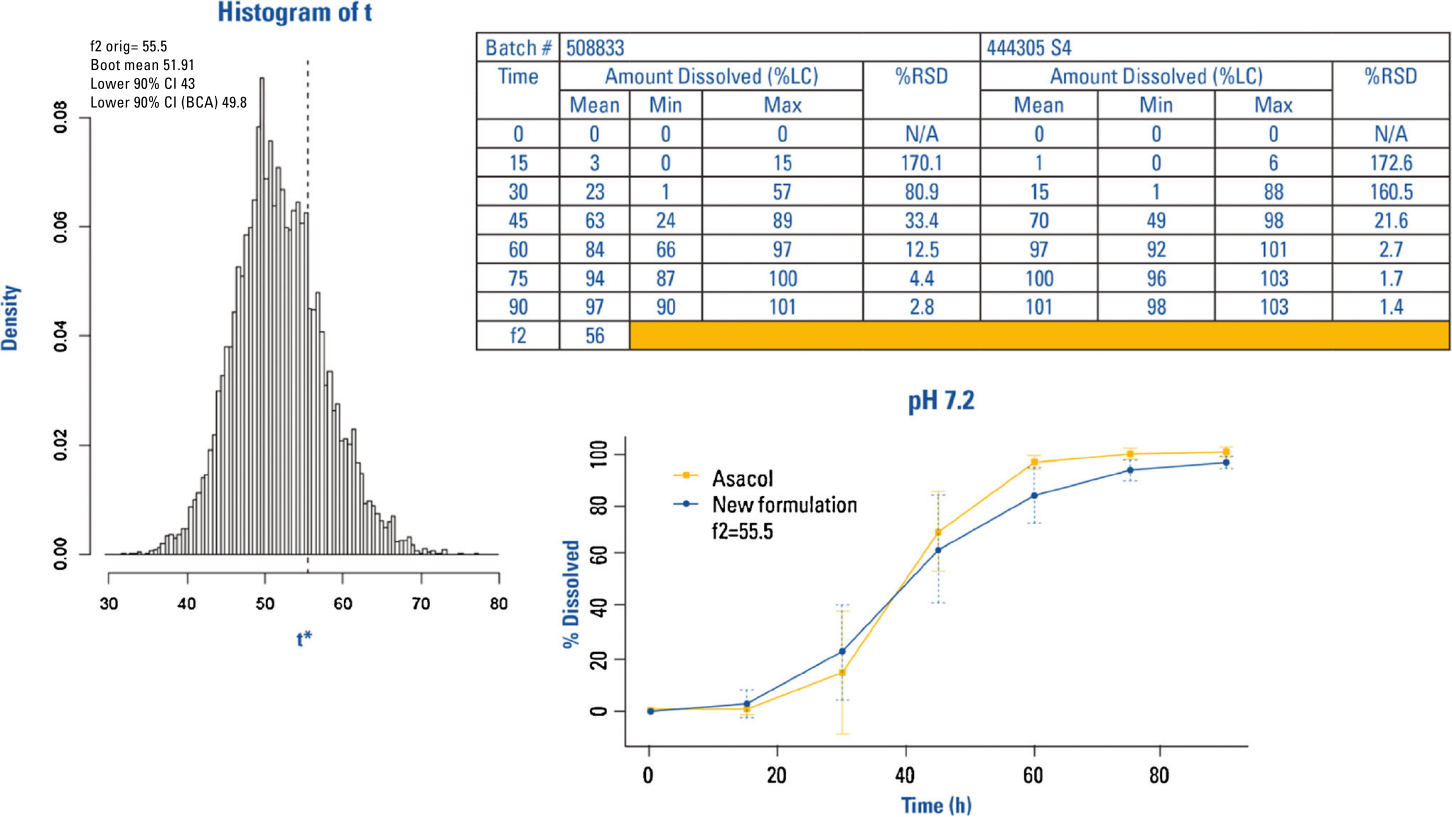
Example: bootstrap versus original method for *f*2 determination results

A multipoint dissolution profile comparison was generated between the mesalamine capsules and tablets over a range of pH values (pH 4.5, 6.0, 6.5, 6.8, 7.2, and 7.5). The similarity factors (*f*2) values were calculated and provided for each dissolution media. Figure 2 displays the dissolution profiles before and after the formulation change at pH at 7.2, for which the *f*2 value was calculated to be 55.5 by the applicant. The variability (%RSD) in the dissolution data was very high at the 15- (170.1%), 30- (80.9%) and 45- (33.4%) min time points. Because of this, the *f*2 test could not be used and the FDA applied the model independent bootstrap approach.

The bootstrap lower confidence interval with a bias correction was equal to 49.8. The FDA accepted this value in demonstrating similarity of the two products. The bootstrap method should not be used to improve an *f*2 but rather it should be used as a mechanism for dealing with the issue of data variability. It is noted that there is an open software available for the bootstrap calculation (13).

### Model Dependent Approaches

The model dependent approach is recommended for the dissolution data which consist of at least four or more dissolution data points. The approach includes the selection of a suitable mathematical function to describe the dissolution data. Several mathematical models have been described in the literature to fit dissolution profiles (2,14,15) including Probit, Logistic, Weilbull, Quadric and Exponential, Hixson-Crowell *k*, and first-order *k*. The ultimate choice for the model is made based on the goodness of fit criteria such as the least residual mean square error and Akaike Information Criteria. Ideally, an acceptable fit shows relatively small differences between the fitted and the observed data, presenting no trends in residual error, and includes a small number of model parameters (a model with no more than three parameters is recommended (2)).

Having a selected mathematic model, additional steps are needed to accomplish the model-dependent approaches (2):

1. Define an appropriate similarity region based on intra- and inter-batch variances of the fitted model for test units from the standard approved batches.
2. Estimate the model parameters by fitting individual units (12 units) of the reference and the test to the chosen mathematical function.
3. Calculate the statistical distance between the mean parameters of the test and reference.
4. Calculate a 90% confidence region around the statistical distance is computed.
5. Compare the limits of the confidence region with pre-defined similarity region to declare either similarity or dissimilarity of the profiles.

Bootstrap methods generally make no assumptions about data distribution; hence, they can be a useful approach when there are too few data to test or verify model assumptions. Disadvantage of the use of the model-dependent approach is that there is no single universally accepted method to evaluate the validity of the statistical assumptions of the model. Examples of model-dependent approaches to characterize *in vitro* dissolution profiles are presented elsewhere (14,15).

The similarity *f*2 test should not be used when an approved *in vitro in vivo* correlation (IVIVC) model is available for the drug product. In this case, the IVIVC model should be used to predict the rate (*C*_max_) and extent (AUC) of systemic exposure based on dissolution profile data of the “test” and “reference” products (before and after the specific proposed CMC change).

## REGULATORY APPLICATIONS OF *F*2

### The Application of *f*2 in QbD

Dissolution and *f*2 testing can be used to link clinical performance to critical manufacturing and process attributes and therefore used to set clinically relevant manufacturing process controls and product specifications that provide assurance of consistent quality product throughout its life cycle.

### Is *f*2 Applicable to All Dosage Forms?

When the drug availability is spontaneous (e.g., inhalation), or when the drug release rate is preprogrammed (e.g., implants), statistical tests of the dissolution profile similarity may not adequately characterize *in vivo* product performance. In these cases, other test procedures may be necessary (e.g., aerodynamics, particle size distribution, diffusion cell systems, dose counter).

Dosage forms where *f*2 has been shown to be applicable include the following:

- Simple dosage forms: immediate release
- Oral complex dosage forms: modified release (DR, ER) and combination (IR/IR, IR/MR, MR/MR) products
- Non-oral dosage forms, including transdermal drug delivery systems and drug-device combination products

## FREQUENTLY ASKED QUESTIONS

1. If dissolution profile is incomplete can *f*2 be estimated?
  Scenario 1—plateau is not reached: no
  Scenario 2—plateau is reached: yes
2. When is it not necessary to calculate the *f*2 value?
  The product used in pivotal human study is the same as that being commercialized
3. Can the *f2* test be estimated across studies?
  Yes, in some cases, *f*2 can be estimated across studies. It is possible to use *f*2 when the study materials being compared are of the same age (shelf-life) and if the same dissolution method (and detection method) is being used for the analysis, for example, comparing batches that have been manufactured after a process change with product batches that were submitted in an approval. The comparison would be the initial data from the approval batches with the initial data from the changed batches. In the case of clinical trial material being compared to a formulation change prior to marketing, the dissolution profiles can be compared if the dissolution method and detection methods are the same and the age of the product when it was tested is the same as the new product being tested.
4. Can *f*2 be used to evaluate similarity of *in vitro* drug release profiles of non-oral drug-device combination products?
  Yes, if the products are evaluated using the same testing conditions and share identical rate controlling factors for the *in vitro* and *in vivo* drug release.
5. If dissolution is complete in less than 15 min, are there additional considerations that are needed before declaring profile comparability?
  May need additional earlier time points (e.g., 5, 10 min).
  May need to change dissolution testing conditions (harsh to mild).
6. What factors can potentially contribute to variability in dissolution profiles?
  Dissolution method related (as previously discussed).
  Formulation related.
  Analytical method related.

## CONCLUSIONS

When planning the statistical method to be applied for profile comparison, there are several pivotal considerations that need to be integrated into the study design. Model-dependent or independent approaches should be considered to confirm prolife similarity. The variability should be minimized and the release profile should contain at least three time points to be eligible for the *f*2 calculation. When developing a new drug product, it is prudent to plan for dissolution documentation during all phases of drug development so that if a change is needed in formulation, manufacturing process, equipment, or manufacturing site, dissolution profiles and *f*2 data can be used in lieu of the need to conduct PK/clinical trials.

The *f*2 test can be invaluable for evaluating the similarity of the dissolution profiles, thereby supporting product manufacturing and development based upon quality by design, supporting the product and process throughout the design space.
